# Supplementary material for: Somatostatin interneurons activated by 5-HT2A receptor suppress slow oscillations in medial entorhinal cortex
Source: eLife. 2021 Mar 31;10:e66960. doi: 10.7554/eLife.66960 (PMC8016478; doi:10.7554/eLife.66960)
Supplement: Transparent reporting form [file elife-66960-transrepform.pdf]

eLIFE
Westbrook Centre, Milton Road
Cambridge CB4 1YG
UK
P 01223 855340
W elifesciences.org
T @elife

eLife's transparent reporting form

We encourage authors to provide detailed information *within their submission* to facilitate the interpretation and replication of experiments. Authors can upload supporting documentation to indicate the use of appropriate reporting guidelines for health-related research (see EQUATOR Network), life science research (see the BioSharing Information Resource), or the ARRIVE guidelines for reporting work involving animal research. Where applicable, authors should refer to any relevant reporting standards documents in this form.

If you have any questions, please consult our Journal Policies and/or contact us: editorial@elifesciences.org.

Sample-size estimation

- You should state whether an appropriate sample size was computed when the study was being designed
- You should state the statistical method of sample size computation and any required assumptions
- If no explicit power analysis was used, you should describe how you decided what sample (replicate) size (number) to use

Please outline where this information can be found within the submission (e.g., sections or figure legends), or explain why this information doesn't apply to your submission:

Sample size, including number of mice (in vivo) and number of cells (in vitro), was chosen to be similar with previously published studies on the effect of neuromodulation on oscillations as in Vandecasteele M, Varga V, Berényi A, et al. Optogenetic activation of septal cholinergic neurons suppresses sharp wave ripples and enhances theta oscillations in the hippocampus. Proc Natl Acad Sci U S A. 2014;111(37):13535-13540. doi:10.1073/pnas.1411233111 and Mayne EW, Craig MT, McBain CJ, Paulsen O. Dopamine suppresses persistent network activity via D(1) -like dopamine receptors in rat medial entorhinal cortex. Eur J Neurosci. 2013;37(8):1242-1247. doi:10.1111/ejn.12125.

Replicates

- You should report how often each experiment was performed
- You should include a definition of biological versus technical replication
- The data obtained should be provided and sufficient information should be provided to indicate the number of independent biological and/or technical replicates
- If you encountered any outliers, you should describe how these were handled
- Criteria for exclusion/inclusion of data should be clearly stated
- High-throughput sequence data should be uploaded before submission, with a private link for reviewers provided (these are available from both GEO and ArrayExpress)

Please outline where this information can be found within the submission (e.g., sections or figure legends), or explain why this information doesn't apply to your submission:

The number of animals and cells analyzed is reported in the main text or in the figure legend.

eLife Sciences Publications, Ltd is a limited liability non-profit non-stock corporation incorporated in the State of Delaware, USA, with company number 5030732, and is registered in the UK with company number FC030576 and branch number BR015634 at the address Westbrook Centre, Milton Road Cambridge CB4 1YG, UK | March 2019

Statistical reporting

- Statistical analysis methods should be described and justified
- Raw data should be presented in figures whenever informative to do so (typically when N per group is less than 10)
- For each experiment, you should identify the statistical tests used, exact values of N, definitions of center, methods of multiple test correction, and dispersion and precision measures (e.g., mean, median, SD, SEM, confidence intervals; and, for the major substantive results, a measure of effect size (e.g., Pearson's r, Cohen's d)
- Report exact p-values wherever possible alongside the summary statistics and 95% confidence intervals. These should be reported for all key questions and not only when the p-value is less than 0.05.

Please outline where this information can be found within the submission (e.g., sections or figure legends), or explain why this information doesn't apply to your submission:

All statistical tests, including T statistics, Kolmogorov-Smirnov, etc are identified within the manuscript when results are reported. All averages are reported as +- standard error of the mean unless otherwise stated.

(For large datasets, or papers with a very large number of statistical tests, you may upload a single table file with tests, Ns, etc., with reference to sections in the manuscript.)

Group allocation

- Indicate how samples were allocated into experimental groups (in the case of clinical studies, please specify allocation to treatment method); if randomization was used, please also state if restricted randomization was applied
- Indicate if masking was used during group allocation, data collection and/or data analysis

Please outline where this information can be found within the submission (e.g., sections or figure legends), or explain why this information doesn't apply to your submission:

For the in vivo part of the study (Figure 1) mice were randomly allocated between control, fenfluramine or MDMA group. Data from all mice were analyzed with the same pipeline.

Additional data files ("source data")

- We encourage you to upload relevant additional data files, such as numerical data that are represented as a graph in a figure, or as a summary table
- Where provided, these should be in the most useful format, and they can be uploaded as "Source data" files linked to a main figure or table
- Include model definition files including the full list of parameters used
- Include code used for data analysis (e.g., R, MatLab)
- Avoid stating that data files are "available upon request"

Please indicate the figures or tables for which source data files have been provided:

Source data are provided for figure 2, 3, 4 and 5. Source data are either excel files or matlab files with associated code. Analysis was performed in Matlab and Prism. Github links of the repositories used are available on the data availability statement and in the "materials and method" section. A database relative to Figure 1 is available at http://dx.doi.org/10.17632/c4twt96888.1, associated code at: https://github.com/Schmitz-lab/De-Filippo-et-al.-2020
